# What explains the large disparity in child stunting in the Philippines? A decomposition analysis

**DOI:** 10.1017/S136898002100416X

**Published:** 2022-11

**Authors:** Valerie Gilbert T Ulep, Jhanna Uy, Lyle Daryll Casas

**Affiliations:** 1 Philippine Institute for Development Studies, 18F Three Cyberpod Centris – North Tower, EDSA Cor. Quezon Avenue, Quezon City, Philippines; 2 Ateneo Policy Center, School of Government, Ateneo de Manila University, Quezon City, Philippines; 3 Health Sciences Program, School of Science and Engineering, Ateneo de Manila University, Quezon City, Philippines

**Keywords:** Malnutrition, Stunting, Inequality, Philippines, Oaxaca-Blinder

## Abstract

**Objective:**

About one-third of under-five Filipino children are stunted, with significant socio-economic inequality. This study aims to quantify factors that explain the large gap in stunting between poor and non-poor Filipino children.

**Design:**

Using the 2015 Philippine National Nutrition Survey, we conducted a linear probability model to examine the determinants of child stunting and then an Oaxaca-Blinder decomposition to explain the factors contributing to the gap in stunting between poor and non-poor children.

**Setting:**

Philippines.

**Participants:**

1881 children aged 6–23 months participated in this study.

**Results:**

The overall stunting prevalence was 38·5 % with a significant gap between poor and non-poor (45·0 % *v*. 32·0 %). Maternal height, education and maternal nutrition status account for 26 %, 18 % and 17 % of stunting inequality, respectively. These are followed by quality of prenatal care (12 %), dietary diversity (12 %) and iron supplementation in children (5 %).

**Conclusions:**

Maternal factors account for more than 50 % of the gap in child stunting in the Philippines. This signifies the critical role of maternal biological and socio-economic circumstances in improving the linear growth of children.

Stunting is a marker of chronic malnutrition that affects 144 million children and causes significant disease burden worldwide^(1,2)^. Stunting is an important determinant of human capital and is a predictor of economic productivity^(3–5)^. It is linked to poorer cognitive and educational outcomes^(6,7)^, lower wages^(8,9)^, and poorer health in adult life^(3,10)^.

The Philippines is about to become an upper middle-income country, but stunting remains of very high public health significance^(11)^. The average stunting rate in upper middle-income countries is only 14 %, while the Philippines hovers at 30 %, comparable to the poorest countries in the world^(12)^. From 2000 to 2015, the country’s income per capita has increased by 3–4 % annually from 2000 to 2015^(12)^, but stunting prevalence had barely improved during the same period with only a 0–1 % annual decline^(12,13)^. In contrast, many low- and middle-income countries have experienced a large decline even in countries notoriously known to have high burden^(14,15)^. The paradoxical relationship of economic growth and chronic malnutrition in the Philippines was against the backdrop of slow decline in poverty incidence and persistently high-income inequality. From 2000 to 2015, the percentage of the population below the poverty threshold hovered at 21 % to 25 % and the Gini coefficient at 0·46 to 0·42^(16)^.

The high prevalence of child stunting is reinforced by the large disparity across socio-economic status. In 2015, about 36 % of under-five children from the bottom 20 % were stunted compared to 14 % among the richest 20 %^(11)^. This 22-percentage point absolute difference between the poor (bottom 20 %) and non-poor (top 20 %) makes the Philippines one of the countries with the highest gap^(1)^. Globally, the average gap is only 11 percentage points^(17)^. Hence, understanding the drivers of stunting inequality could guide the appropriate design of nutrition and health interventions for the country. While studies have examined the correlates of stunting in the Philippines, the magnitude of the inequality and its drivers are not well-documented^(18–20)^.

This study aims to determine the magnitude of socio-economic inequality in stunting among 6–23 months Filipino children and to decompose the inequality into maternal, health and nutrition and socio-demographic factors. We used a nationally representative sample from the 2015 Philippine National Nutrition Survey (NNS).

## Methods

### Data

We used the 2015 Philippine NNS, a cross-sectional nationally representative survey covering all 17 regions and 80 provinces of the country. The NNS is the official data source on nutritional status, diet and other lifestyle-related risk factors in the Philippines. The survey employed a stratified three-stage sampling. The first stage of the sampling was the selection of the primary sampling unit which consisted of one village with at least 500 households each. From these sampling units, housing units were randomly selected from enumeration areas with 150–200 households. In the last stage, households were randomly selected. In total, 42 310 households with 17 702 children aged 0–60 months were sampled in the survey. All members of the household, including children, were then included to participate^(11)^. We requested the expanded microdata from the Food and Nutrition Research Institute (FNRI). The details of the survey can be obtained here: http://enutrition.fnri.dost.gov.ph/site/uploads/2015_OVERVIEW.pdf.

Our analytical sample is 1881 children aged 6–23 months with complete records for anthropometric, socio-demographic, maternal and health data. We focused on the 6–23 age group only because this is the period when the sharp divergence in stunting prevalence occurs and the gap remained steady from 24 to 60th month. The figure in Appendix A shows the acceleration of the height-for-age *Z*-score between Q1 (poorest) and Q5 (richest), which starts at 6 months until 24 months.

### Variables and measurements

#### Dependent variable

Height-for-age is the dependent variable of interest. In the 2015 NNS, the height of children under-2 years of age was measured in a recumbent position using a medical plastic infantometer and the standing height of those 2 years and above was measured using a stadiometer following the standard procedures. We estimated the height-for-age *Z*-score using the 2006 WHO Child Growth Standards. We categorised a child as stunted if their height-for-age *Z*-score is two sd below the median^(21)^.

#### Socio-economic variable

We measured socio-economic status using a wealth index predicted from principal component analysis. The index is based on the ownership of wide-range assets (e.g. television, radio, refrigerator)^(22,23)^. We categorised households as ‘poor’ if they belonged to the bottom 40 % in the wealth distribution otherwise non-poor. We considered the bottom 40 % of the wealth distribution as poor because it captures the portion of the population living below or just above estimated national poverty threshold. It also captures the prevalence of moderate and severe food insecurity, which is around 54 % in 2015^(11)^.

#### Independent variables

**Fig. 1:**
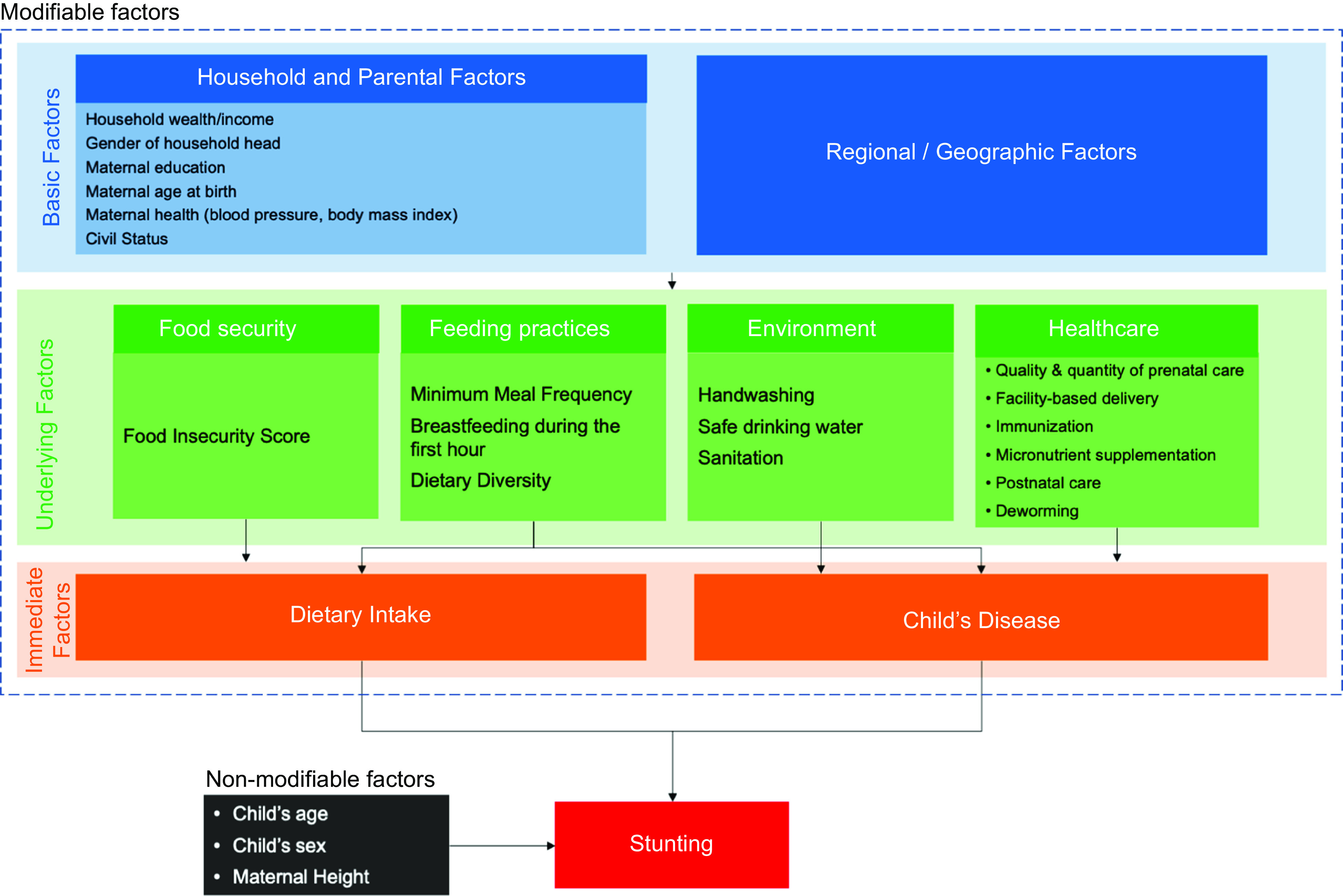
Conceptual Framework. Source: adapted from Rizal MF and van Doorslaer (2018)

To identify the independent variables to be included in our model, we adopted a framework from UNICEF (1990), which was refined by Fikru-Rizal and van Doorslaer (2019)^(24)^ (See Fig. 1). Under this framework, determinants of stunting are divided into non-modifiable and potentially modifiable factors. Non-modifiable factors include child age and sex^(25)^. Mother’s height was also considered as a non-modifiable factor because some part of a child’s height is explained by genetic factors^(26)^. Modifiable factors were categorised as basic, underlying and immediate factors. Immediate factors, that is, dietary intake and child’s disease (orange box) are conduits by which the underlying determinants such as food insecurity, feeding practices, environment and healthcare services (green box) and the basic factors such as household and parental factors and regional or geographic factors affect the child nutritional status (red box).

Under household factors, we included the following variables: sex of the household head, maternal education, age (in years) and maternal BMI, as well as maternal blood pressure. We categorised maternal education into binary high school and non-high school graduate, and BMI using WHO cut-offs for underweight, normal, overweight and obese^(27)^. For the maternal blood pressure, we categorised it into systolic blood pressure above and below 140 mmHg, since the cut-off for high blood pressure based on the 2020 Clinical Practice Guidelines on hypertension in the country^(28)^.

Under underlying factors, we included food insecurity, feeding practices, environment and healthcare factors. We measured food insecurity using a score predicted from principal component analysis of five (5) food security-related questions that were categorised into high, medium and low food insecurity. Under feeding practices, we considered three variables: minimum meal frequency (MMF) and Dietary Diversity Score (DDS). MMF, a proxy indicator for a child’s energy requirements, examines the number of times children received foods other than breast milk^(29)^. MMF is met if a breastfed and non-breastfed children 6–23 months of age receive solid, semi-solid or soft foods or milk feed the minimum number of 4 times per day or more^(29)^. DDS is an indicator on the quality of diet of the child. The DDS was defined as the number of unique food groups consumed by the child the previous day^(30)^. Under environmental factors, we considered water, sanitation and hygiene variables such as handwashing practices, garbage disposal and availability of safe drinking water and sealed toilet facilities. Under healthcare factors, we determined the quality score using factor analysis using variables pertaining to services conducted during prenatal care such as anthropometric measurement, blood pressure treatment/diagnosis, blood test, urinalysis, ultrasound, micronutrient supplementation, tetanus toxoid and nutrition counseling; quality scores were categorised into low, medium and high. We determined whether the mother had postnatal care after giving birth, and whether the child was born in a health facility, and whether she had received three doses of DPT vaccine, received iron supplement, vitamin A supplement and had ever been dewormed. Appendix B outlines the operational definitions of independent variables.

#### Data analysis

We started with bivariate analyses by examining the distribution of dependent and independent variables by socio-economic status. We used *t*-test to determine the significant difference in stunting and other independent variables between poor and non-poor Filipino children. After bivariate analyses, we performed two (2) inferential statistics. First, we conducted a linear probability model (LPM) to examine the determinants of stunting. LPM is an ordinary least square regression using a binary dependent variable (that is, 1 = stunted; 0 = non-stunted). In our regression model, we controlled food insecurity, feeding practices, environment, healthcare factors, household and other non-modifiable factors outlined in Fig. 1. A coefficient in an LPM is interpreted as the change in the probability that Y = 1 (i.e. stunted) for a one-unit change of the independent variable of interest, holding everything else constant. We chose LPM over logistic regression model because it is computationally tractable and easier to interpret the coefficients^(31)^.

Second, we decomposed the difference in stunting prevalence between poor and non-poor children using the Oaxaca-Blinder method, an econometric tool used in labour economics to examine differential wage gaps between groups (e.g. by sex, race, residence)^(32)^. Oaxaca-Blinder has been used to assess disparities in health outcomes and healthcare utilisation expenditures^(33–39)^. The decomposition aims to quantify the contribution of selected predictors in explaining the gap in the prevalence of stunting between poor and non-poor children. The gap is decomposed into three parts: the first part is known as explained or endowment effect (E) and claimed the gap due to differences in the distribution of determinants between poor and non-poor; the second part is the unexplained or coefficient effect (C) and claimed the gap due to the differences in the effect of determinants between the groups and the third is an interaction between both—endowment effect and coefficient effect (CE)^(40)^. The intuition behind Oaxaca Blinder decomposition can be described by the subsequent equations.

The first step is to estimate a linear regression for both poor, *p* and non-poor, *np* children. The *y*, is the outcome variable, that is,i.e. stunted (2 sd below median). *X*’s are a vector of explanatory variables as listed in Appendix B.(1)
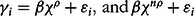




The gap between the mean outcomes, *γ^np^*, and *γ^p^* is equal to:(2)
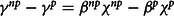

where *χ^np^* and *χ^p^* are vectors of explanatory variables evaluated at the means for the non-poor and poor, respectively. Assuming exogeneity, the error terms in Equation 1 are zero. Estimates of difference in the gap in mean outcomes were obtained by substituting sample means of the Xs and estimates of the parameter on *β* in Equation 1:(3)
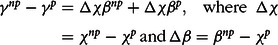




The differences in Xs are weighted by the coefficients of the poor group and the differences in the coefficients are weighted by the Xs of the non-poor group; thus, partitioning the gap in outcomes between two groups:(4)
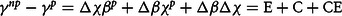

Whereas, the gap in outcome was from a gap in endowments (E), a gap in coefficients (C) and a gap arising from the interaction of endowments and coefficients (CE).

We conducted all our analysis using STATA 15 software, with statistical significance determined at *P* ≤ 0·05. We accounted for the complex design of the survey using provided survey weights in the microdata from Food and Nutrition Research Institute (FNRI).

#### Ethical standards disclosure

We used public use files of the DOST-FNRI NNS that was conducted according to the guidelines laid down in the Declaration of Helsinki, and all procedures involving research study participants were approved by the FNRI Institutional Ethics Review Committee (FNRIEC). Written informed consent was obtained from all subjects and their participation was strictly voluntary and they were allowed to withdraw their participation at any time without any consequence. The NNS data were available publicly, and the study participants were anonymous and thus did not require ethics approval.

## Results

**Fig. 2:**
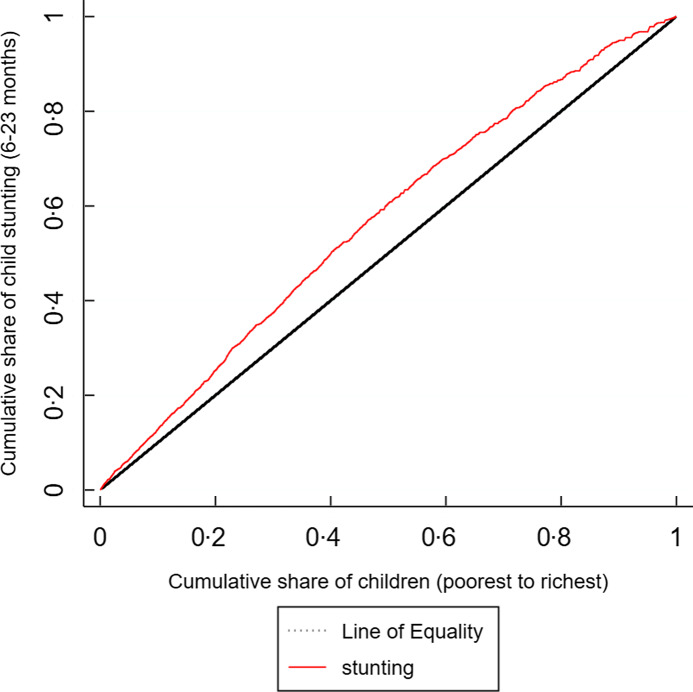
Concentration curve presenting the degree of socio-economic inequality in stunting among children aged 6–23 months. Source: authors’ analysis of 2015 National Nutrition Survey

We included 1881 children with complete records for anthropometric, socio-demographic, maternal and health data aged 6–23 months in our analysis. Overall, 38·5 % of the children were stunted. The prevalence of stunting among Filipino children belonging to poor households (45 %) was significantly higher compared to non-poor children (32 %). Using a concentration curve to display disparity, Fig. 2 presents the degree of inequalities in stunting among 6–23 months children. The area below the 45° line of equality represents a progressive concentration curve, and the area below represents a regressive concentration curve. Given that the curve is above the diagonal, the stunting ‘burden’ in the Philippines is concentrated more heavily in the poor.

Table 1 shows the results of bivariate analyses between socio-economic status and dependent and independent variables. Children from poor households have lower probability to have a mother with at least high school education, mother with postnatal care after giving birth, to be food secure, to meet MMF and diversity, to have access to safe drinking water and improved toilet, to have timely and high-quality prenatal care, to have facility-based delivery, to have complete DPT vaccine and to have iron supplementation.

**Table 1:** Description of sample of children 6–23 months of age

Variable	Category	All	Poor	Non-poor	*P*-value
Stunted	% Stunted	38·5	45·0	32·0	0·00*
Sex of child	% Female	52·3	52·8	51·8	0·76
	% Male	47·7	47·2	48·2	0·76
Child’s age	Average age in months	17·7	17·7	17·7	0·98
Maternal height	Average height in cm	151·5	150·6	152·3	0·25
Civil status	Single	9·0	7·7	9·9	0·08
	Married	89·0	90·8	87·6	0·08
	Separated/widowed	2·0	1·5	2·5	0·08
Female as household head	% Female	1·8	1·9	1·6	0·45
Maternal education	% Below high school	38·8	58·1	23·6	0·00*
	% High school graduate and above	61·2	41·9	76·4	0·00*
Maternal age at birth	Average age in years	30·1	30·6	29·8	0·02*
High systolic blood pressure of mother	% with 140 mmHg or above	2·7	3·2	2·3	0·21
	% below 140 mmHg	97·3	96·8	97·7	0·21
Maternal BMI	% Underweight	13·6	15·0	12·5	0·13
	% Normal	46·5	51·3	42·8	0·00*
	% Overweight	28·0	25·7	29·6	0·06
	% Obese	11·8	7·9	14·8	0·00*
Food insecurity score	% Low	24·6	10·9	33·3	0·00*
	% Medium	34·8	33·5	35·7	0·00*
	% High	40·5	55·6	31·0	0·00*
Minimum meal frequency (MMF)	% meeting MMF	94·0	91·6	95·6	0·00*
	% not meeting MMF	6·0	8·4	4·4	0·00*
Dietary diversity score (DDS)	Average DDS	3·2	2·9	3·3	0·00*
Breastfeeding within the first hour of life	% Yes	65·9	68·4	64·0	0·09
Handwashing before preparing the food of the child	% Never	4·0	4·3	3·8	0·53
	% Always	89·4	88·4	90·1	0·53
	% Sometimes	6·6	7·3	6·1	0·53
Dispose garbage by dumping or throwing	% Yes	18·8	25·9	13·3	0·00*
Availability of safe drinking water	% Yes	61·9	47·1	73·4	0·00*
Availability of toilet (categorical)	% None	9·6	20·2	1·3	0·00*
	% Yes, water sealed	84·6	70·7	96·0	0·00*
	% Yes, not sealed	5·8	9·1	2·7	0·00*
Post-natal care	% Yes	91·4	85·8	95·4	0·00*
Timely prenatal care	% Yes	78·2	75·4	80·5	0·00*
Quality of prenatal care	% Low	32·7	44·4	23·7	0·00*
	% Medium	28·5	30·1	27·2	0·00*
	% High	38·8	25·5	49·1	0·00*
Place of delivery	% Home	21·2	33·1	12·0	0·00*
	% Government hospital	37·8	32·6	42·8	0·00*
	% Government clinics	12·7	17·4	9·1	0·00*
	% Private hospital/clinic	28·1	17·0	36·8	0·00*
Complete DPT vaccine	% Yes	58·9	54·8	62·1	0·00*
Iron supplementation in children	% Yes	19·4	15·1	22·8	0·00*
Vitamin A supplementation in children	% Yes	68·9	71·5	66·8	0·03*
Deworming	% Yes	60·7	66·7	56·1	0·00*
Geographical regions	Region I	4·6	3·4	5·3	0·00*
	Region II	4	3·4	4·3	0·00*
	Region III	10·2	4·3	14·1	0·00*
	Region IV-A	12·4	5·9	16·5	0·00*
	Region V	6·3	8·3	5	0·00*
	Region VI	8·7	12	6·6	0·00*
	Region VII	7·2	8·3	6·5	0·00*
	Region VIII	5	8·2	2·9	0·00*
	Region IX	3·8	5·4	2·8	0·00*
	Region X	4·6	5·8	3·8	0·00*
	Region XI	4·8	6·7	3·6	0·00*
	Region XII	4·8	6·8	3·6	0·00*
	NCR	11·6	3·8	16·7	0·00*
	CAR	1·6	1·6	1·6	0·00*
	ARMM	5·1	9	2·6	0·00*
	Region XIII	3	3·6	2·6	0·00*
	MIMAROPA	2·3	3·5	1·5	0·00*

* *P* < 0·05.

Table 2 shows the coefficients from the LPM, which is interpreted as the change in stunting for every one-unit change of the independent variable, *ceteris paribus.* Among non-modifiable and maternal factors, child’s sex, maternal height, maternal education and maternal nutrition status were significantly associated with stunting. Being a female decreases the probability of being stunted by 11 percentage points, and one (1) centimeter increase in maternal height decreases the probability of being stunted by 2 percentage points. Mothers without high school education increase their probability by 6 percentage point difference). Mothers with normal BMI are less likely to have stunted children compared to underweight (11 %) and overweight (16 %) counterparts.

**Table 2:** OLS regression coefficients using linear probability model

Variables	Coefficient	se	95 % CI	
			Lower limit	Upper limit
Female sex	−0·11*	0·02	−0·15	−0·06
Child’s age	0·06	0·04	−0·01	0·13
Child age squared	0·00	0·00	0·00	0·00
Maternal Height	−0·02*	0·00	−0·03	−0·02
Civil status married (Ref: single)	0·02	0·04	−0·05	0·10
Civil status separated/widowed	0·17*	0·10	0·01	0·36
Female household head	0·05	0·10	−0·14	0·24
Maternal education below high school	0·06	0·03	0·01	0·11
Maternal age at birth	0·00	0·00	0·00	0·00
Maternal high blood pressure	0·00	0·06	−0·11	0·12
BMI normal (Ref: Underweight)	−0·11*	0·03	−0·18	−0·05
BMI overweight	−0·16*	0·04	−0·24	−0·09
BMI obese	−0·20*	0·04	−0·29	−0·11
Food insecurity medium (Ref: low food insecurity)	−0·05	0·03	−0·11	0·01
Food insecurity high	−0·01	0·03	−0·07	0·05
Minimum meal frequency (MMF)	−0·15*	0·05	−0·24	−0·05
Dietary diversity score (DDS)	−0·02*	0·01	−0·04	−0·01
Breastfeeding within the first hour	0·01	0·02	−0·04	0·05
Handwashing always (Ref: never)	0·08	0·05	−0·02	0·19
Handwashing sometimes	0·03	0·06	−0·10	0·16
Garbage disposal	−0·01	0·03	−0·06	0·05
Safe drinking water	−0·04	0·02	−0·08	0·00
Toilet facility sealed (Ref: No)	−0·03	0·04	−0·10	0·04
Toilet facility not sealed	0·09	0·05	−0·01	0·20
On time prenatal care	−0·04	0·03	−0·09	0·01
Quality of prenatal care medium (Ref: low)	−0·02	0·03	−0·07	0·03
Quality of prenatal care high	−0·06*	0·03	−0·12	−0·01
Place of delivery government hospital (Ref: home)	−0·27	0·33	−0·92	0·04
Place of delivery government clinic	0·14	0·39	−0·06	0·09
Place of delivery private facility	−0·39	0·36	0·11	0·03
Complete DPT vaccine	−0·03	0·02	−0·07	0·02
Iron supplementation in children	−0·06*	0·03	−0·11	−0·01
Vitamin supplementation in children	−0·02	0·02	−0·07	0·03
Post-natal care	−0·04	0·04	−0·12	0·04
Deworming	0·00	0·03	−0·05	0·05
High SES	−0·01	0·03	−0·06	0·04
Regions: Cagayan Valley (Region II) (ref: Region I)	0·02	0·07	−0·12	0·16
Central Luzon (Region III)	−0·05	0·06	−0·16	0·07
Bicol Region (Region V)	0·00	0·06	−0·13	0·12
Western Visayas (Region VI)	0·03	0·06	−0·08	0·15
Central Visayas (Region VII)	−0·02	0·06	−0·14	0·10
Eastern Visayas (Region VIII)	0·08	0·07	−0·06	0·21
Zamboanga Peninsula (Region IX)	−0·01	0·07	−0·15	0·13
Northern Mindanao (Region X)	0·02	0·07	−0·12	0·15
Davao Region (Region XI)	−0·05	0·07	−0·19	0·09
SOCCSKSARGEN (Region XII)	−0·06	0·07	−0·20	0·07
National Capital Region (NCR)	0·05	0·06	−0·06	0·17
Cordillera Administrative Region (CAR)	0·04	0·09	−0·15	0·22
Autonomous Region in Muslim Mindanao (ARMM)	0·06	0·07	−0·08	0·20
CARAGA (Region XIII)	0·06	0·08	−0·09	0·21
CALABARZON (Region IV-A)	0·03	0·06	−0·08	0·14
MIMAROPA	0·00	0·08	−0·16	0·17
_cons	3·74	0·43	2·90	4·58

OLS, ordinary least squares, *t* statistics in parentheses.

* *P* < 0·05.

In terms of child feeding practices, MMF and DDS were significantly associated with child stunting. Children meeting their MMF decreases the probability of being stunted by 15 percentage points than children not meeting MMF, and one (1) unit increase in DDS decreases the probability of being stunted by 2 %. In terms of healthcare access, only high-quality prenatal care and iron supplementation were associated with stunting. Mothers having high-quality prenatal decreases the probability of their child being stunted by 6 % than those having low-quality prenatal care. On the other hand, children receiving iron supplementation decrease the probability of being stunted by 6 % than those who are not. We did not observe a significant association between stunting and other healthcare variables such as a place of delivery, postnatal care, vitamin A supplementation in children and DPT immunisation. Neither handwashing, type of toilet nor garbage dumping were significant.

Table 3 shows the results of the Oaxaca-Blinder decomposition model. It shows that children belonging to poor households had higher prevalence of stunting (45 %) than those belonging to non-poor households (32 %), a 13-percentage point absolute gap. The mean difference in stunting rates between the groups was significant (*P* value < 0·05). This gap accounted mostly for the explained part (about 82 %). The unexplained and interactions components contribute to the rest, but the unexplained and interaction terms are not statistically significant. Because of the lack of significance of the two parts, we will only present the endowment effect (E) of the gap.

**Table 3:** Summary result of Oaxaca decomposition analysis showing the mean differences in stunting rates

	coefficient	sd	*P*-value	% contribution
Mean prediction high (H)	0·45	0·02	0·00	
Mean prediction low (L)	0·32	0·01	0·00	
Raw differential (R) {H-L}	0·13	0·02	0·00	
due to endowments or explained (E)	0·11	0·02	0·00	82 %
due to unexplained (C)	0·01	0·03	0·76	7 %
due to interaction (CE)	0·02	0·03	0·59	12 %

**Fig. 3:**
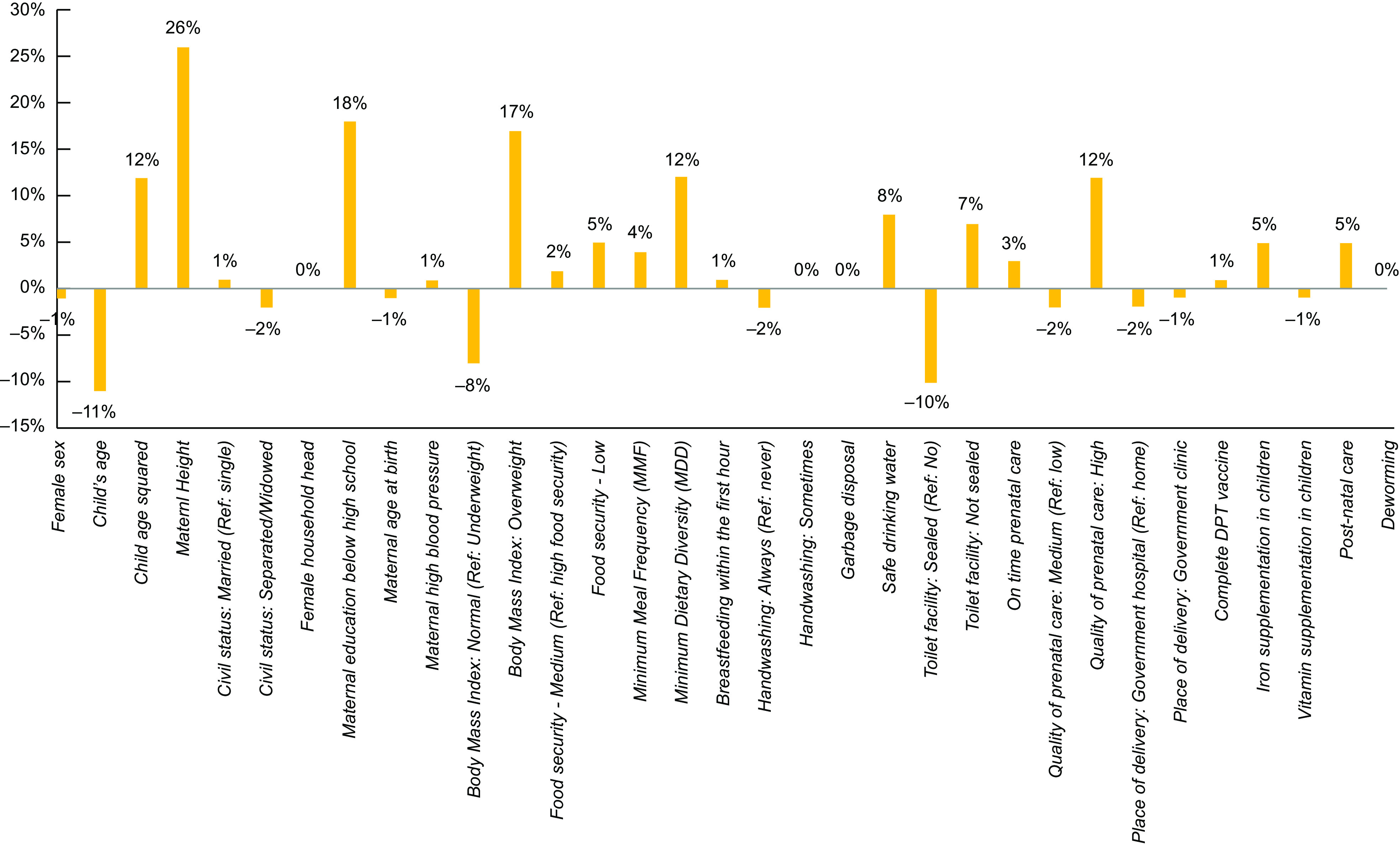
Contributions of each determinant to stunting inequality. Source: Authors’ analysis of 2015 National Nutrition Survey

Table 4 shows how differences in the distribution of each determinant contributed separately to the first part of the gap (endowment effect). In particular, maternal education, height and BMI, iron supplementation in children, quality prenatal care and DDS were the significant contributors explaining the gap in stunting among children between the poor and non-poor. Maternal height contributed the highest, at 26 %, of the gap for stunting followed by maternal education, at 18 %, maternal BMI at 17 %, quality of prenatal care at 12 %, dietary diversity at 12 % and iron supplementation for children at 5 % (See Fig. 3).

**Table 4:** Contribution of each factor in poor and non-poor differentials in stunting (endowments or explained component)

Variable	Coef.	*P*-value	95 % lower limit	95 % upper limit	% share to the gap
Female sex	0·00	0·56	0·00	0·00	−1 %
Child’s age	−0·01	0·42	−0·04	0·02	−11 %
Child age squared	0·01	0·36	−0·01	0·04	12 %
Maternal height	0·03***	0·00	0·02	0·04	26 %
Civil status married (Ref: single)	0·00	0·67	0·00	0·00	1 %
Civil status separated/widowed	0·00	0·19	−0·01	0·00	−2 %
Female household head	0·00	0·63	0·00	0·00	0 %
Maternal education below high school	0·02***	0·04	0·01	0·04	18 %
Maternal age at birth	0·00	0·76	0·00	0·00	−1 %
Maternal high blood pressure	0·00	0·47	0·00	0·00	1 %
BMI normal (Ref: Underweight)	−0·01	0·05	−0·02	0·00	−8 %
BMI overweight and obese	0·02***	0·00	0·00	0·03	17 %
Food insecurity medium (Ref: low food insecurity)	0·00	0·22	0·00	0·01	2 %
Food insecurity high	0·01	0·56	−0·01	0·02	5 %
Minimum meal frequency (MMF)	0·00	0·18	0·00	0·01	4 %
Dietary diversity score	0·01***	0·01	0·00	0·02	12 %
Breastfeeding within the first hour	0·00	0·36	0·00	0·00	1 %
Handwashing always (Ref: never)	0·00	0·36	−0·01	0·00	−2 %
Handwashing sometimes	0·00	0·69	0·00	0·00	0 %
Garbage disposal	0·00	0·92	−0·01	0·01	0 %
Safe drinking water	0·01	0·25	−0·01	0·02	8 %
Toilet facility sealed (Ref: No)	−0·01	0·52	−0·04	0·02	−10 %
Toilet facility not sealed	0·01	0·24	−0·01	0·02	7 %
On time prenatal care	0·00	0·20	0·00	0·01	3 %
Quality of prenatal care medium (Ref: low)	0·00	0·24	−0·01	0·00	−2 %
Quality of prenatal care high	0·01***	0·04	0·00	0·03	12 %
Place of delivery government hospital (Ref: home)	0·00	0·48	−0·01	0·00	−2 %
Place of delivery government clinic	0·00	0·69	−0·01	0·01	−1 %
Complete DPT vaccine	0·00	0·70	0·00	0·01	1 %
Iron supplementation in children	0·01***	0·03	0·00	0·01	5 %
Vitamin supplementation in children	0·00	0·34	0·00	0·00	−1 %
Postnatal care	0·01	0·41	−0·01	0·02	5 %
Deworming	0·00	0·95	−0·01	0·01	0 %

*** *P* < 0·05.

## Discussion

Child stunting in the Philippines is reinforced by the large inequality. We found a significant difference in the prevalence of poor and non-poor Filipino children during the critical period of 6–23 months (45·0 % *v*. 32·0 %; *P* value: 0·000). This gap in stunting prevalence accelerated sharply after the sixth month, which may be explained by the following factors.

In this study, we quantified wide range of socio-economic, maternal and child health factors that could explain the large gap in stunting prevalence between poor and non-poor children. Maternal education, height, and BMI, quality of prenatal care, diversity of child’s diet and iron supplementation for children were the significant contributors to the large disparity between poor and non-poor. Among the factors included in our decomposition model, maternal factors (that is, maternal nutrition and maternal education) account for more than 50 % of the gap. This suggests the role of maternal circumstance in perpetuating the large disparity in chronic malnutrition in the Philippines.

Maternal education explains about 18 % of the gap in child stunting. Social and cognitive factors could explain the role of education in the disparity of child stunting. Firstly, mothers with higher levels of education have more access to health information hence they have more knowledge of optimal child feeding and rearing practices. Secondly, education increases social capital^(41)^. If the child is ill or malnourished, the mother has access to information from her networks on how to treat the child. Thirdly, higher levels of education provide skills that are socially valued and give women a higher status, which raises self-confidence and eases social interaction with other high-status actors including health workers^(42)^. Fourthly, mothers with higher levels of education are associated with employment and income, which increases their demand for health and nutrition services^(43–45)^.

Maternal height explains about 26 % of the gap. While genetics plays an important role, non-genetic factors largely explain the disparity^(46)^. In poor settings, maternal height is a proxy for maternal nutrition practices^(47)^. Mothers with shorter stature are more likely to be poor and living in a constrained environment, hence complementary feeding is typically suboptimal^(48)^. Short maternal height, which is more prevalent among the poor, also leads to low birth weight and eventually child stunting^(49–51)^. Mothers with shorter stature have decreased macronutrient and energy stores, and smaller reproductive organs, which limit fetal growth *in utero* leading to low birthweight^(48)^.

BMI explains about 17 % of the gap. Empirical studies consistently show the association between maternal BMI and child stunting^(52–55)^. The pathway in which underweight status affects subsequent anthropometric failure of the child starts *in utero*. The intergenerational transmission of maternal underweight gives infants a higher risk of low birth weight which is a manifestation of early age undernutrition, that may progress to childhood undernutrition^(55,56)^. In the Philippines, about 11 % of adult women were considered underweight, with large disparity across socio-economic status. The large percentage of poor women who are underweight reflects the limited access to high-quality and adequate diet and food supplement especially during pregnancy (i.e. balanced energy protein supplementation)^(11)^.

At the same time, maternal overnutrition is also found to be associated as a risk factor contributing to the gap in stunting. Previous nutritional history of the mother may explain this because studies show that children who also experienced chronic malnutrition (i.e. stunting) at an early age may have an impact on their physiologic and metabolic characteristics that result in having higher chances of being overweight during adulthood^(57,58)^. An emerging phenomenon-that should also be examined is the presence of a double burden of malnutrition, wherein undernutrition and overnutrition coexist in the same household. Nutrition transition may also be related to this, wherein there is a change in lifestyle, dietary patterns and physical activity associated with economic developments^(59,60)^.

The quality of prenatal care explains 12 % of the gap. During prenatal care visits, mothers are given prenatal advice about child feeding and rearing practices, and they are provided with appropriate health and nutrition services such as micronutrient supplementation^(61)^. Poor mothers are less likely to have access to high-quality prenatal care in primary care facilities because of financial and physical barriers^(62–64)^. Although prenatal care services are subsidised by the Philippine government, other indirect expenses come with utilizing the service such as transportation expenses and other medications and supplements, which are not provided for free^(62,65,66)^. In the Philippines, more than 50 % of Filipinos seeking outpatient care use out-of-pocket financing^(67)^.

DDS explain 12 % of the gap. Dietary diversity is an important component of dietary quality. Consumption of different food items and food groups is associated with improved nutritional adequacy of the diet^(68,69)^. A high DDS increases the density of complimentary food, which is critical in ensuring optimal growth and development for the child^(70,71)^. Consistent with other studies, poverty is associated with low DDS^(72–74)^. This reflects challenges on food insecurity in the country and poor knowledge on optimal child feeding and rearing practices^(73,75)^. In the Philippines, 75 % of the poor (bottom 40 %) are moderate to severely food insecure^(11)^, and 39 % of mothers do not have correct knowledge about the duration of complementary feeding^(11)^.

Iron supplementation contributes to around 5 % of the gap. Provision of iron supplements was found to be associated with linear growth based on some evidence^(76–79)^. Iron deficiency may lead to anaemia and may contribute to growth retardation especially in poor families^(78)^. Iron deficiency anaemia is characterised by low haemoglobin concentration in the blood. This happens when the iron stores are utilised and depleted following the lack of intake of iron from food or supplements^(77,80)^. Poor children have limited access to iron supplements because of geographical and financial barriers in accessing primary care facilities, which is the entry point of health and nutrition interventions including micronutrient supplementation^(81,82)^. In the Philippines, data from 2013 shows that among children under-five, prevalence of anaemia was the highest among the poorest (16·5 %) and lowest among the richest (7·9 %)^(83)^.

Our findings are found to be consistent with studies conducted in other countries. Studies in Bangladesh, Ethiopia, India, Iran and Tanzania all reported that socio-economic disparity in stunting in their countries is observed, having higher stunting prevalence among the poor than their non-poor counterparts^(84–88)^. Maternal factors, such as nutrition status (height and BMI) and maternal education, were consistent drivers of the socio-economic gap in stunting across the studies, parallel to our findings. Kumar and Singh (2013) noted the limited use of maternal health care services serves as the main driver of inequality, thus recommending access to these services to reduce the gap^(86)^.

The sharp acceleration of the gap in stunting prevalence between poor and non-poor after the sixth month is also consistent with other studies^(89,90)^. The nutritional requirement of the child increases after the period of exclusive breastfeeding (sixth month)^(91)^; hence, those children coming from poor households were not able to cope as a function of a wide range of socio-economic, maternal and child health factors mentioned above (e.g. lack of access to quality prenatal care and healthcare services, diverse diet, food insecurity).

Reducing the gap in stunting requires interventions aimed to improve socio-economic circumstances of poor women and their access to essential health and nutrition services. This involves collaboration of different sectors such as education, health, social welfare, agriculture, etc. Reducing the drop-out rate in secondary education among the poor women can be improved through provision of innovative approaches (e.g. expansion of conditional cash transfers)^(92–94)^. Teenage pregnancy is one of the common reasons why students drop-out of school. Hence, the provision of modern contraceptives to young adolescents should be explored^(95–98)^. Mothers with formal education will be equipped with better knowledge on achieving optimal nutrition for her and their child^(99,100)^ and will have opportunities for better jobs and income, which will be a function of their capability to access essential health and nutrition services (e.g. prenatal care)^(101–103)^. Access to high-quality health and nutrition services can be improved by providing social protection among poor women and their families, through the expansion of social health insurance benefits and coverage^(104–106)^. The primary care system should be strengthened because it serves as the initial point of contact of individuals and families to the health system, giving access to vast essential health and nutrition services such as nutrition education for appropriate child feeding and rearing practices, balanced-energy protein supplementation for the mother and vitamin and mineral supplementation (e.g. iron supplements) beneficial for both the mother and the child. Currently, the Philippine Health Insurance Corporation, the country’s national health insurance, does not provide comprehensive primary care benefits.

In this study, we are able to use survey data that is nationally representative, which gives us a clear picture of the determinants of the stunting inequality in the country. Unlike other studies, which examined only a few determinants, our analysis examined a wide range of maternal and child health and socio-demographic indicators in our decomposition^(85,87)^. There are limitations in the design and methods of the study. Our analytical sample focused only on a small number of children (*n* 1881) because we did not include those children aged 24–60 months and those children without complete anthropometric, socio-demographic, maternal and health data. For the measurement of socio-economic status, we used a proxy indicator of wealth index, which may not be able to account for the size and consumption of the household, and may not be able to capture income disruption^(107)^. We are only able to measure the effects of relevant variables in the data, but the disparity could be influenced by other factors such as child’s exposure to illnesses, food/dietary intake and birth weight.

## Conclusion

We have identified the factors that explain the large disparity in stunting between poor and non-poor Filipino children. Maternal factors (i.e. maternal education, maternal height and maternal nutrition status) account for more than 50 % of the inequality in child stunting. This reinforces the critical role of maternal socio-economic circumstances in improving the linear growth of children in addition to expanding the service coverage and quality of essential nutrition and health interventions such as prenatal care and appropriate complementary feeding.

## Supplementary material

For supplementary material accompanying this paper visit https://doi.org/10.1017/S136898002100416X. click here to view supplementary material
