# Supplementary material for: What explains the large disparity in child stunting in the Philippines? A decomposition analysis
Source: Public Health Nutr. 2021 Oct 4;25(11):2995–3007. doi: 10.1017/S136898002100416X (PMC9991861; doi:10.1017/S136898002100416X)
Supplement: Supplementary file 1 [file S136898002100416Xsup001.docx]

**Appendices**


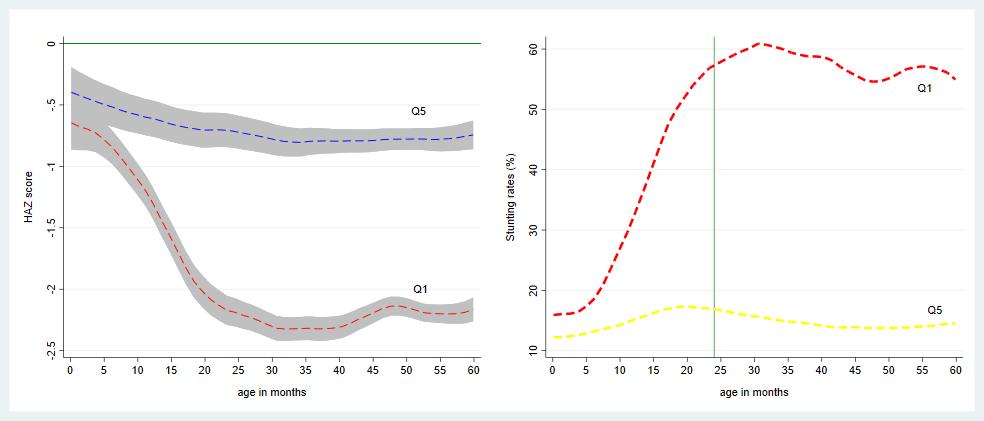


**Appendix A**. Height-for-age z-score (HAZ), by age and socio-economic status

Source: Author’s analysis of 2015 National Nutrition Survey

**Appendix B. Operational definition of independent variables**

| Domain | Variable | Category | Operational definition |
| --- | --- | --- | --- |
| Non-modifiable factors | | | |
| Non-modifiable factors | Sex of child (binary) | Female |  |
|  |  | Male |  |
|  | Child’s age (continuous) | Age in months |  |
|  | Maternal height (continuous) | Height in centimeters |  |
| Modifiable factors | | | |
| **Basic Factors** | | | |
| Household and parental factors | Gender of household head (binary) | Female |  |
|  |  | Male |  |
|  | Maternal education (binary) | High school undergraduate or below |  |
|  |  | High school graduate and above |  |
|  | Maternal age at birth (continuous) | Age in years |  |
|  | Systolic blood pressure (binary) | 140mmHg or above |  |
|  |  | Below 140mmHg |  |
|  | Maternal Body Mass Index (ordinal) | Underweight |  |
|  |  | Normal |  |
|  |  | Overweight |  |
|  |  | Obese |  |
|  | Civil Status | Single |  |
|  |  | Married |  |
|  |  | Separated/Widowed |  |
| Underlying Factors | | | |
| Food insecurity | Food insecurity score of the household (categorical) | High food insecurity | The food security (FD) scores were categorized into tertiles (3). FD scores were predicted using Principal Component Analysis of five (5) food security-related questions. |
|  |  | Medium food insecurity |  |
|  |  | Low food insecurity |  |
| Feeding practices | Minimum Meal Frequency (binary) | Yes | Breastfed and non-breastfed children 6-23.9 months of age who receive solid, semi-solid, or soft foods or milk feed the minimum number of times or more. |
|  |  | No |  |
|  | Breastfeeding during the first hour | Yes |  |
|  |  | No |  |
|  | Dietary Diversity Score (count) | 0-7 | Number of food groups consumed by the child the previous day. |
| Environment | Handwashing before preparing the food of the child (categorical) | No |  |
|  |  | Always |  |
|  |  | Sometimes |  |
|  | Dispose garbage by dumbing or throwing (binary) | Yes |  |
|  |  | No |  |
|  | Availability of safe drinking water | Yes |  |
|  |  | No |  |
|  | Availability of toilet (categorical) | None |  |
|  |  | Yes, water sealed |  |
|  |  | Yes, not sealed |  |
| Healthcare services | Timely prenatal care (binary) | Yes | Mothers with on time (within the first trimester) first prenatal check-up during their pregnancy. |
|  |  | No |  |
|  | Quality of prenatal care (categorical) | Low | The quality scores were categorized into tertiles (3). Prenatal quality scores were predicted using Principal Component Analysis (PCA) using variables pertaining to services conducted during prenatal care: weight and height measurement, blood pressure treatment/diagnosis, blood test, urinalysis, ultrasound, micronutrient supplementation, tetanus toxoid, and nutrition counselling. |
|  |  | Medium |  |
|  |  | High |  |
|  | Place of delivery (categorical) | Home |  |
|  |  | Government hospital |  |
|  |  | Government clinics |  |
|  |  | Private hospital/clinic |  |
|  | Complete DPT vaccine | Yes |  |
|  |  | No |  |
|  | Iron supplementation in children | Yes |  |
|  |  | No |  |
|  | Vitamin A supplementation in children | Yes |  |
|  |  | No |  |
|  | Post-natal care | Yes |  |
|  |  | No |  |
|  | Deworming | Yes |  |
|  |  | No |  |
